# A case-control study of trace-element status and lung cancer in Appalachian Kentucky

**DOI:** 10.1371/journal.pone.0212340

**Published:** 2019-02-27

**Authors:** Jason M. Unrine, Stacey A. Slone, Wayne Sanderson, Nancy Johnson, Eric B. Durbin, Shristi Shrestha, Ellen J. Hahn, Fran Feltner, Bin Huang, W. Jay Christian, Isabel Mellon, David K. Orren, Susanne M. Arnold

**Affiliations:** 1 Department of Plant and Soil Sciences, University of Kentucky, Lexington, KY, United States of America; 2 Department of Toxicology and Cancer Biology, University of Kentucky, Lexington, KY, United States of America; 3 Markey Cancer Center, University of Kentucky, Lexington, KY, United States of America; 4 Department of Preventative Medicine and Environmental Health, University of Kentucky, Lexington, KY, United States of America; 5 Department of Epidemiology, University of Kentucky, Lexington, KY, United States of America; 6 Kentucky Cancer Registry, University of Kentucky, Lexington, KY, United States of America; 7 Department of Internal Medicine, University of Kentucky, Lexington, KY, United States of America; 8 BREATHE, College of Nursing, University of Kentucky, Lexington, KY, United States of America; 9 Center of Excellence in Rural Health, University of Kentucky, Hazard, KY, United States of America; 10 Department of Biostatistics, University of Kentucky, Lexington, KY, United States of America; Chinese Academy of Sciences, CHINA

## Abstract

Appalachian Kentucky (App KY) leads the nation in lung cancer incidence and mortality. Trace elements, such as As, have been associated with lung cancers in other regions of the country and we hypothesized that a population-based study would reveal higher trace element concentrations in App KY individuals with cancer compared to controls. Using toenail and drinking water trace element concentrations, this study investigated a possible association between lung cancer incidence and trace-element exposure in residents of this region. This population-based case-control study had 520 subjects, and 367 subjects provided toenail samples. Additionally, we explored the relationship between toenail and fingernail trace-element concentrations to determine if fingernails could be used as a surrogate for toenails when patients are unable to provide toenail samples. We found that, contrary to our initial hypothesis, trace element concentrations (Al, As, Cr, Mn, Co, Fe, Ni, Cu, Se, and Pb) were not higher in cancer cases than controls with the exception of Zn where concentrations were slightly higher in cases. In fact, univariate logistic regression models showed that individuals with lower concentrations of several elements (Al, Mn, Cr, and Se) were more likely to have lung cancer, although only Mn was significant in multivariate models which controlled for confounding factors. While drinking water concentrations of Al, Cr and Co were positively related to cancer incidence in univariate models, only Co remained significant in multivariate models. However, since the drinking water concentrations were extremely low and not reflected in the toenail concentrations, the significance of this finding is unclear. We also found that fingernail concentrations were not consistently predictive of toenail concentrations, indicating that fingernails should not be used as surrogates for toenails in future studies.

## Introduction

Exposure to potentially carcinogenic trace-elements can occur as a result of both occupational and environmental exposures. As, Cd, Ni and other trace elements have been consistently, but to varying degrees, linked to lung cancer in ecologic, cohort, and case-control studies [1–3]. For example, a previous study in New England linked low to moderate levels of exposure to As with the development of small cell and squamous cell forms of lung cancer [4], and a recent report linked low level As exposure with female lung cancer [3]. However, Ferdosi and his colleagues [5] found no association with lung cancer rates and As concentrations in groundwater, so further study in a high cancer incidence region is warranted. Exposure to trace elements occurs through groundwater contamination, ingestion of local food and wild game, exposure to soil and airborne particulates, and through occupations such as metal-working, leather tanning, and mining [6–8].

The Commonwealth of Kentucky, particularly southeastern Appalachian Kentucky (App KY), has a high incidence of chronic diseases; mortality rates from lung cancer are 54% higher for men and 39% for women than the U.S. national average. Data from the Kentucky Cancer Registry (KCR), a Surveillance, Epidemiology and End Result (SEER) site, reveals the age-adjusted incidence rate for lung cancer in App KY was 109.1 per 100,000 residents from 2010–2014, compared to 53.1 per 100,000 nationally [9, 10]. We have established that smoking rates alone may not explain the elevated lung cancer incidence for App KY [11]. Other areas of the state have similar smoking rates, but significantly lower lung cancer incidence [9, 10]. App KY is an area of intensive surface coal mining which has occurred from the 1700’s to present day and which has the potential to increase exposures to trace elements [12]. We hypothesized that exposure to trace elements, either through environmental or occupational exposure routes and likely in combination with smoking, plays a role in the observed elevation in lung cancer prevalence and designed this population-based case-control study to evaluate this hypothesis.

Toenail concentrations have been validated as a biomarker of trace element exposure [13]. They are advantageous because they integrate exposure over several months while blood or urine concentrations often only reflect exposure over days [14]. As compared to fingernails, toenails also grow at slightly less than half the rate of fingernails and therefore integrate a longer period of exposure [15]. Bones and teeth integrate trace element exposure over a much longer duration; however, collection of these samples is too invasive for living subjects. Many of the trace-elements of concern also concentrate in keratin, which is rich in sulfhydryl binding sites. As compared to hair, nail tissue has a higher percentage of keratin and is more efficient at concentrating trace elements and is a better choice for cancer patients undergoing chemotherapy, who may be bald [14].

Our previous ecological study suggested that toenail concentrations of As, Cr and Ni were higher for residents of App KY as compared to residents of Jefferson County, an urbanized county in northwestern KY with a lower lung cancer rate [16]. Given the results of this ecological study, we designed a comprehensive case-control study of trace element concentrations in App KY participants with no history of lung cancer (controls) or those diagnosed with lung cancer (cases). App Ky also has the highest lung cancer incidence in the U.S., which should maximize the detection of a difference between cases and controls. Our objective was to test the hypothesis that patients with lung cancer had higher trace element exposures than controls. To accomplish this, we tested and compared trace element concentrations, primarily in toenails, from cases and controls, and also tested water samples from their homes. As a second objective of this study, we aimed to examine the correlation between fingernail and toenail concentrations, as it is not always possible to collect toenails, due to missing limbs, digits, or nails; or the existence of foot conditions such as diabetes mellitus, fungal infections, or other conditions. While a previous study compared mean fingernail and toenail concentrations of trace elements for a relatively small sample (n = 50) of healthy volunteers, to our knowledge, the correlation between toenail concentrations and fingernail concentrations has not been examined on a large and heterogeneous sample set [14]. If fingernail concentrations could reliably predict toenail concentrations, then they could be used as a substitute in future studies from patients that cannot provide toenail samples.

## Methods, patients and materials

### Eligibility

This study was approved by the University of Kentucky Institutional Review Board, approval number 45179. Informed consent was obtained in writing from all study participants. From January 9, 2012 to August 20, 2014, subjects were enrolled into this study. Eligibility for enrollment included: 1) residence in southeastern Kentucky (defined as 5^th^ Congressional District) at the time of enrollment, 2) a working phone, 3) English speaking, 4) age greater than 17, and 5) for cases: no prior history of other cancers (other than stage I and II non-melanomatous skin cancer, or synchronous primary within 6 months) and for controls: no prior history of cancer by self-report.

### Subject enrollment procedures

Controls were selected using stratified random sampling from voter registration rolls of the 29 counties in the 5^th^ Congressional District. A frequency matching method was used to match control and case in 4:1 ratio by gender and age (± 5 years). Controls were selected prior to the knowledge of gender and age of recruited cases, in order to facilitate recruitment and these estimates were based on the gender and age distribution of lung cancer cases in the 5^th^ District in year 2009 and 2010. Following the first six months of recruitment, the Cancer Prevention and Control (CPC) staff compared the distributions of recruited controls and cases and the response rates of control and cases, and randomly selected further cohorts of controls to properly match the distribution of recruited cases. It should be noted that App KY is not racially or ethnically diverse, with over 96% of residents identifying as White, non-Hispanic; however, consistent with the Belmont Report [17], all races and or ethnicities were eligible for inclusion. Control subjects were contacted via phone by CPC staff and screened for eligibility and consent for home visit.

#### Rapid case ascertainment and participant recruitment

All newly diagnosed and histologically or cytologically confirmed lung cancer cases (International Classification of Diseases-9^th^ revision, 162.2–162.9) [18] were identified by the KCR for inclusion. Because the median survival time for all lung cancer patients is very short, recruitment procedures utilized the innovative rapid case ascertainment system developed by the KCR. All Kentucky health care facilities are required to report all cancer cases using the Cancer Patient Data Management System (CPDMS) developed by KCR [19]. This method uses the electronic pathology (e-path) system to provide the KCR with immediate notification of a cancer diagnosis each time a pathology report is generated. KCR served as the “honest broker” of the private health information for these patients. To identify each newly diagnosed lung cancer patient in the 5th District, a certified tumor registrar (CTR) at KCR weekly reviewed all of the incoming e-path reports indicating a diagnosis of lung cancer. KCR then sent a letter to the physicians for each eligible lung cancer patient to determine whether any issues (i.e. dementia, frailty) would preclude the patient from study participation (passive consent). Eligible patients were mailed a letter asking for their assent for contact by Kentucky Homeplace, a well-established lay health worker program in App KY. Patients who did not respond to the letter within two weeks were called by KCR staff to obtain their assent.

#### Home visits

KCR staff created eligible study lists for contact by trained Kentucky Homeplace field research associates (FRA) to administer the questionnaire and collect biospecimens and drinking water samples from cases and controls at their homes. Additional screening of cancer patients was performed by Patient Navigators/FRAs at the Center for Rural Health in Hazard, Kentucky; St. Claire Regional Medical Center, Morehead, Kentucky; and the University of Kentucky. The FRAs were trained phlebotomists who performed all study procedures in subjects’ homes. Prior to a home visit by the research team, subjects gave verbal assent by phone and before data and sample collection began, study volunteers were provided a description of the study. FRAs obtained written informed consent and Health Insurance Portability and Accountability Act (HIPAA) authorization approved by the Institutional Review Board (IRB) of the University of Kentucky and regional partner institutions.

### Demographic and health data collection

Participants underwent an in-person interview using a standardized questionnaire to identify potential risk factors. This included demographics, lifetime history of tobacco usage, estimates of second-hand tobacco smoke exposure, occupational history, work history (industry, job title, duties, and time period of employment), diet, exercise, family, and medical histories. With the exception of the residential history portion of the questionnaire, all other sections of the questionnaire were validated in the Centers for Disease Control and Prevention (CDC), National Health and Nutrition Examination Survey (NHANES), National Health Interview Survey, and Behavioral Risk Factor Surveilance System (BRFSS)[20–22]. The entire questionnaire was pilot-tested with volunteers from App KY to ensure that the questions were relevant and applicable to the test group. To remain focused on the hypotheses of this study, and maintain adequate statistical power, not all variables collected were included in the analysis presented here. In particular, we did not include second-hand tobacco smoke exposure, occupational and work history, exercise or family and medical histories.

### Nail and drinking water sample collection, preparation and analysis

Where possible, at least 50 mg dry mass nails were collected from all digits on both hands and feet using pre-cleaned and sterilized surgical-grade stainless steel clippers and stored in polyethylene bags until preparation and analysis. Methods were based on those used by Heck et al. [4]. Prior to analysis, the nails were transferred to 1.8 mL polypropylene centrifuge tubes which were filled trace metal grade acetone (Fluka 69508) and ultrasonicated for 30 min in a bath-type ultrasonic cleaner to remove organic contaminants and nail polishes. If any visible polish remained for a sample the process was repeated. One sample was rejected because the polish could not be removed. The samples were then rinsed thrice with 18.2 MΩ cm^-1^ resistivity deionized water (DI). The process was then repeated with a 1% v/v aqueous solution of Citranox detergent (an acidic detergent designed to remove superficial metal contamination; Sigma-Aldrich Z273236). Samples were then dried at 60°C to a constant mass and then transferred to trace-metal free 15 mL polypropylene centrifuge tubes (VWR 89049–170). To digest the samples, we added 0.75 mL of ultra-pure concentrated HNO_3_ (Aristar-Ultra, VWR 87003–658) and 0.25 mL of concentrated H_2_O_2_, heated them to 100°C in 30 min and held them at that temperature for 10 min using a temperature-controlled microwave reaction system (CEM MARS Xpress, Mathews, NC, USA). The samples were then brought to 15 mL with DI prior to analysis. We included reagent blanks and certified reference material (CRM) samples with every digestion set. The CRM was number 13 (human hair; National Institute of Environmental Studies, Tsukuba, Japan). There is no certified reference material available for nails, and being made of keratin, hair is the closest match. Because no subsampling was performed, digestion replicates were not prepared.

Drinking water samples were collected in metal free polypropylene vials and acidified to pH < 2 with ultra-pure HNO_3_. The samples were collected from the main tap used for drinking water consumption (typically the kitchen). The tap water was allowed to run for approximately 5 minutes prior to sample collection to flush water from the plumbing system. We did not remove screens or aerators from the taps. We periodically collected method blanks by filling the sample vials with DI water at the homes where the samples were collected. The CRM used was standard reference material (SRM) 1643e, trace elements in water (National Institute of Standards and Technology, Gaithersburg, MD, USA).

Analysis of trace-elements was performed using an inductively coupled plasma mass spectrometer equipped with an octopole reaction system (ICP-MS; Agilent 7500cx, Santa Clara, CA, USA). We used standard mode for aluminum (Al), manganese (Mn), iron (Fe), cobalt (Co), nickel (Ni), copper (Cu), zinc (Zn), arsenic (As), cadmium (Cd), lead (Pb) and uranium (U), pressurized the reaction cell with hydrogen gas (H_2_) for Fe and Se to remove argon oxide ion (ArO^+^) and argon chloride ion (ArCl^+^) interferences, and pressurized the reaction cell with helium (He) for Cr to remove argon carbide ion (^40^A^12^C^+^) interferences. An internal standard mixture containing 1 μg/L of scandium (Sc), germanium (Ge), yttrium (Y), indium (In), terbium (Tb) and bismuth (Bi) was mixed with the samples online in a solution of 1% v/v concentrated ultra-pure nitric acid (HNO_3_) and 5% v/v butanol. The butanol was included to normalize C concentrations among the samples and standards since C causes positive interferences for Se and Cr. Calibration was performed using a certified reference standard (Inorganic Ventures, Christiansburg, VA, USA). Validity of the calibration curve was assessed by analyzing a standard from the same source but with a different lot number after every 10 samples and after every calibration. The calibration curve was considered valid if the observed concentration for the independent standard was within 10% of the expected concentration. We also determined spike recovery on randomly selected samples during each analytical run.

### Hair collection and nicotine analysisμ

To determine if Se concentrations in toenails were correlated with current smoking levels, hair samples (approximately 15–20 strands) were collected from the back of the scalp by a trained field research staff. Samples were immediately placed in paper envelopes and stored at room temperature. Reversed phase high-performance liquid chromatography with electrochemical detection (HPLC-ECD) was used to analyze the hair samples for nicotine [23]. The method detection limit was 0.05 μg nicotine/g hair.

### Radon detection and analysis

Residential exposure to radon was measured using long-term alpha track radon detectors. The radon detectors were deployed in the lowest livable level of each study home by field research staff who recorded the specific location of the detector and the mailer box as well as the date when testing began. Study participants were reminded using an automated system 90 days later to stop testing, place the detector in the mailer box, and send via mail to the laboratory (RSSI, Morton Grove, IL, USA) for analysis.

### Statistical analyses

For those with reportable toenail samples, a descriptive analysis was performed to examine the demographics, clinical, and environmental factors by case/control status and those who did not provide samples versus those who did. Continuous demographic variables were summarized by median and range values and compared by Wilcoxon rank sum. Fisher’s exact test were used for comparisons of categorical variables. Correlations among trace-element concentrations in toenails were examined using a Pearson’s correlation matrix. We used logistic regression models to relate trace element concentrations with cancer incidence. Since controls were selected by frequency matching on age and gender, age and gender were included in all logistic regression models. For the multivariate models, covariates which were significant in the bivariate analysis were added, including BMI, education, household income and pack years, were added along with all of the trace element concentrations into a stepwise selection model. All trace-element concentrations were included in all models and not subject to stepwise selection. Only covariates were subject to stepwise selection. Age, pack years, and BMI were adjusted as continuous random variables, while education and household income were categorical variables. The results from the final model which includes all covariates with p-values less than 0.1 are reported. To relate trace element concentrations with dietary variables, we used multiple linear regression and included smoking status, age, sex and BMI as co-variates. Some of the trace element analyses were below the method detection limit (MDL) given either due to a small mass of toenail provided by the subject or low trace element concentration. Several approaches were tested to impute the cases with below the MDL and the results were similar. The final decision was to impute the values of cases with MDL with half of the MDL values [5]. All statistical analyses were performed in SAS 9.3 4 and all statistical tests are two-sided with a significance level of 0.05.

## Results

### Participant recruitment and demographic data

Out of 2,776 lung cancer cases contacted by KCR, 27.7% (770) agreed to be contacted by the project personnel, and 5.4% (150) were recruited. Out of 10,915 randomly selected controls, 19.4% (2,115) agreed to be contacted by the project and 3.4% (370) were recruited in the study. Demographics of the full cohort are described in **S1 Table**. A total of 367 (100 cases and 267 controls) subjects provided toenail samples and 317 of those subjects also provided fingernail samples. Demographics for subjects who provided toenail samples compared between cases and controls are shown in **Table 1**. Full cohort demographics compared between cases and controls are shown in **S1 Table**. Full cohort demographics compared betwen subjects who provided toenail samples versus those who didn’t is shown in **S2 Table.** Subjects providing toenail samples were significantly younger (62 vs 65 years, median), more likely to be female (56.9% vs 47.7%) and more likely to have higher education and income levels than subjects who didn’t. Within the 367 subjects who provided toenail samples, there were no significant differences in age, sex or race between cases and controls. There was a significant difference (*p =* 0.004) in body mass index (BMI) calculated using a body mass from 3 months prior to study entry. Cases had a median BMI of 26.7 and controls median BMI of 28.7, possibly due to weight loss due to their illness and associated therapy, or appetite suppressant effects of smoking. As expected, there was also a significant difference in smoking status (*p <* 0.0001) with 51.0% of cases being current smokers as compared to 19.1% of control. The median time at current residence was 26 years (IQR: 13–40 years) with only 5% having been in the same residence for less than 5 years. Data showing numbers of subjects contacted and recruited by gender, age, and case/control status are shown in **S3 Table.**

**Table 1:** Cohort demographics broken down by case and control for those who provided nails.

		Case	Control	All	p-value
N		100	267	367	
Age		62.0 (39.0–89.0)	62.0 (26.0–91.0)	62.0 (26.0–91.0)	0.99
BMI (3 mos ago)		26.7 (14.6–55.2)	28.7 (18.1–66.6)	28.5 (14.6–66.6)	0.004
Created: Comorbidity Score		2.0 (0.0–11.0)	1.0 (0.0–11.0)	1.0 (0.0–11.0)	0.001
Pred fruits/vegs incl legumes & fries c/day		2.5 (1.6–3.7)	2.6 (1.6–4.2)	2.5 (1.6–4.2)	0.03
Pred fruits/vegs incl legumes & no fries c/day		2.3 (1.4–3.5)	2.4 (1.4–4.1)	2.4 (1.4–4.1)	0.03
Pred fruits c/day		0.8 (0.4–2.2)	0.9 (0.4–2.0)	0.8 (0.4–2.2)	0.31
Pred vegetables incl legumes & fries c/day		1.6 (1.1–2.6)	1.7 (1.1–2.7)	1.7 (1.1–2.7)	0.04
Pred vegs incl legumes & no fries c/day		1.5 (1.0–2.5)	1.6 (0.9–2.5)	1.5 (0.9–2.5)	0.03
Pred dairy c/day		1.5 (0.9–3.4)	1.4 (0.9–4.3)	1.5 (0.9–4.3)	0.23
Pred total added sugars tsp/day		15.4 (10.0–36.0)	14.7 (10.0–32.2)	14.9 (10.0–36.0)	0.27
Pred added sugars from sugar-sweetened bevs tsp/d		6.3 (3.6–21.9)	5.3 (3.6–20.8)	5.6 (3.6–21.9)	0.01
Pred whole grains oz/day		0.6 (0.3–1.8)	0.8 (0.3–1.8)	0.8 (0.3–1.8)	0.03
Pred fiber gm/day		15.6 (11.1–25.5)	16.8 (11.3–25.0)	16.4 (11.1–25.5)	0.005
Pred calcium mg/day		889.8 (632.2–1483.9)	911.7 (652.8–1797.5)	906.6 (632.2–1797.5)	0.17
Gender	Female	61 (61.0%)	148 (55.4%)	209 (56.9%)	0.34
	Male	39 (39.0%)	119 (44.6%)	158 (43.1%)	
Race	White	98 (98.0%)	262 (98.1%)	360 (98.1%)	1.00
	Non-White	2 (2.0%)	5 (1.9%)	7 (1.9%)	
Education		1 (1.0%)	0 (0.0%)	1 (0.3%)	<0.0001
	8th Gr or Less	20 (20.0%)	19 (7.1%)	39 (10.6%)	
	Grade 9–11	19 (19.0%)	17 (6.4%)	36 (9.8%)	
	HS Educ	29 (29.0%)	98 (36.7%)	127 (34.6%)	
	College Educ	31 (31.0%)	133 (49.8%)	164 (44.7%)	
Household Income		2 (2.0%)	0 (0.0%)	2 (0.5%)	<0.0001
	<$15K	38 (38.0%)	34 (12.7%)	72 (19.6%)	
	$15K-<$25K	28 (28.0%)	42 (15.7%)	70 (19.1%)	
	$25K-<$50K	16 (16.0%)	75 (28.1%)	91 (24.8%)	
	$50K-<$75K	5 (5.0%)	41 (15.4%)	46 (12.5%)	
	$75K +	4 (4.0%)	47 (17.6%)	51 (13.9%)	
	RTA/DK	7 (7.0%)	28 (10.5%)	35 (9.5%)	
Smoker	Current	51 (51.0%)	51 (19.1%)	102 (27.8%)	<0.0001
	Former	46 (46.0%)	78 (29.2%)	124 (33.8%)	
	Never	3 (3.0%)	138 (51.7%)	141 (38.4%)	
Diabetes	No	79 (79.0%)	224 (83.9%)	303 (82.6%)	0.27
	Yes	21 (21.0%)	43 (16.1%)	64 (17.4%)	
COPD	No	56 (56.0%)	232 (86.9%)	288 (78.5%)	<0.0001
	Yes	43 (43.0%)	34 (12.7%)	77 (21.0%)	
	DK	1 (1.0%)	1 (0.4%)	2 (0.5%)	
Chronic Lung Disease	No	34 (34.0%)	180 (67.4%)	214 (58.3%)	<0.0001
	Yes	66 (66.0%)	87 (32.6%)	153 (41.7%)	
Chronic Heart Disease	No	67 (67.0%)	206 (77.2%)	273 (74.4%)	0.05
	Yes	33 (33.0%)	61 (22.8%)	94 (25.6%)	

RTA = refused to answer, DK = didn’t know, pred = predicted, mos = months, vegs = vegetables, inc = including, c/day = cups (240 mL) per day, tsp = teaspoon (5 mL), bevs–beverages, gr = grade, educ = education, K = thousand, COPD = chronic obstructive pulmonary disease. Numbers in parenthesis indicate the range of values for age and BMI and percentages for count data.

### Toenail concentrations

An MDL was calculated for each individual sample and was a function of the variation in the background signal (3σ) for reagent blanks and the sample mass of each individual sample. Several elements had observations below the MDL for >5% of the samples. The median MDL ranged from 0.006 μg/g for U and Co to 1.21 μg/g for Fe (**Table 2**). U and Cd were excluded from further analysis because 94% and 83%, respectively, of the observations were below the MDL. As, Pb and Co had 18%, 28% and 15%, respectively, of observations below the method detection limit. Measured values for the human hair CRM (CRM Number 13) were generally in good agreement with the certified or reference values with the exception of Al and Fe, which were 49 and 71% of reference values, respectively (**S4 Table**). To obtain good recovery for these elements, stabilization with HCl is often required [24]; however, use of HCl would have created an unacceptable polyatomic interference for As (arsenic chloride ion;^35^Cl^40^Ar^+^), a key element of interest.

**Table 2:** Median method detection limits (MDLs) by element.

Element	Median MDL (μg/g)
Arsenic (As)	0.011
Chromium (Cr)	0.100
Nickel (Ni)	0.083
Cadmium (Cd)	0.045
Lead (Pb)	0.009
Zinc (Zn)	0.130
Uranium (U)	0.006
Iron (Fe)	1.210
Aluminum (Al)	0.323
Manganese (Mn)	0.027
Cobalt (Co)	0.007
Copper (Cu)	0.035
Selenium (Se)	0.014

Note that MDL for each individual sample depended on sample mass.

There were significant moderate correlations between concentrations of several elements in toenails (**Table 3**). The strongest correlations were among Cr, Ni, Co, Mn and Fe. Median trace element concentrations in cancer cases were either similar or lower than in controls, except for Zn, where concentrations were 10% higher than controls (**Table 4**). Because the data were log-normally distributed, it is also informative to examine the 90^th^ percentiles, which can be different even if the medians are similar. The 90^th^ percentiles for Cr, Al, Se, Ni, Cu and Mn were lower for cancer cases than controls, while the 90^th^ percentiles for Fe and Zn were higher. To analyze for differences in the probability of being a case or control as a function of toenail concentration, we employed logistic regression models. The odds ratios and associated *p* values are presented in **Table 5**. The odds ratios indicate the increase or decrease in the odds of being a case per unit increase in toenail trace element concentration. Univariate logistic regression models, which adjusted for age and gender per the matching algorithm, found that as concentrations of Cr, Al, Se and Mn increased, the odds of being a cancer case decreased (**Table 5**). However, in multivariate regression, when also taking into account BMI, and smoking status, only Mn was found to be significant, with the odds of being a cancer case decreasing with increasing Mn concentration (**Table 5**). Multivariate models which included radon activity measured at the subjects homes were also considered, but the results were similar. Although there was some moderate correlation between concentraitons of different trace-elements, there were no obvious collinearity effects on model selection.

**Table 3:** Pearson correlation matrix for log of trace element concentrations in toenails.

	Cr	Ni	Pb	Zn	Fe	Al	Mn	Co	Cu	Se
**As**	-0.01 0.8206	-0.10 0.0529	0.40 < .0001	0.21 < .0001	0.23 < .0001	0.23 < .0001	0.21 < .0001	0.16 0.0026	0.22 < .0001	0.07 0.1770
**Cr**	1.00	0.69 < .0001	-0.03 0.5559	-0.04 0.4525	0.17 0.0010	0.22 < .0001	0.64 < .0001	0.60 < .0001	0.06 0.2779	0.12 0.0244
**Ni**	0.69 < .0001	1.00	-0.07 0.2102	0.00 0.9773	0.21 < .0001	0.24 < .0001	0.58 < .0001	0.63 < .0001	0.09 0.0771	0.10 0.0600
**Pb**	-0.03 0.5559	-0.07 0.2102	1.00	0.03 0.6043	0.38 < .0001	0.33 < .0001	0.27 < .0001	0.20 0.0001	0.27 < .0001	0.04 0.4393
**Zn**	-0.04 0.4525	0.00 0.9773	0.03 0.6043	1.00	0.12 0.0220	-0.01 0.8461	-0.00 0.9470	0.01 0.8045	0.42 < .0001	0.42 < .0001
**Fe**	0.17 0.0010	0.21 < .0001	0.38 < .0001	0.12 0.0220	1.00	0.57 < .0001	0.56 < .0001	0.27 < .0001	0.33 < .0001	0.10 0.0444
**Al**	0.22 < .0001	0.24 < .0001	0.33 < .0001	-0.01 0.8461	0.57 < .0001	1.00	0.49 < .0001	0.28 < .0001	0.29 < .0001	0.12 0.0244
**Mn**	0.64 < .0001	0.58 < .0001	0.27 < .0001	-0.00 0.9470	0.56 < .0001	0.49 < .0001	1.00	0.65 < .0001	0.24 < .0001	0.06 0.2265
**Co**	0.60 < .0001	0.63 < .0001	0.20 0.0001	0.01 0.8045	0.27 < .0001	0.28 < .0001	0.65 < .0001	1.00	0.13 0.0155	0.06 0.2384
**Cu**	0.06 0.2779	0.09 0.0771	0.27 < .0001	0.42 < .0001	0.33 < .0001	0.29 < .0001	0.24 < .0001	0.13 0.0155	1.00	0.28 < .0001
**Se**	0.12 0.0244	0.10 0.0600	0.04 0.4393	0.42 < .0001	0.10 0.0444	0.12 0.0244	0.06 0.2265	0.06 0.2384	0.28 < .0001	1.00

Shown are *r* values (top), and *p-*value (bottom).

**Table 4:** Median and 90^th^ percentile toenail trace element concentrations (μg/g dry mass) for lung cancer cases compared to controls.

element	percentile	case	control
As	50th	0.04	0.04
	90th	0.13	0.12
Cr	50th	0.93	1.17
	90th	2.29	3.80
Ni	50th	0.70	0.89
	90th	2.37	3.01
Pb	50th	0.04	0.04
	90th	0.20	0.18
Zn	50th	117	109
	90th	163	147
Fe	50th	11.79	12.03
	90th	29.13	26.82
Al	50th	6.38	7.30
	90th	13.77	18.67
Mn	50th	0.27	0.32
	90th	0.63	0.92
Co	50th	0.02	0.02
	90th	0.05	0.05
Cu	50th	3.09	3.25
	90th	5.01	5.06
Se	50th	0.90	0.94
	90th	1.17	1.26

Percentiles broken down by case/control, age and smoking status are presented in **S5 Table**.

**Table 5:** Logistic regression results for trace elements in toenails.

	Model					
	Univariate			Multi-variate		
Trace Element	OR	95% CI	p	OR	95% CI	p
Arsenic	1.032	(0.80, 1.34)	0.811	.		.
Chromium	0.701	(0.55, 0.89)	0.004	.		.
Nickel	0.921	(0.77, 1.10)	0.366	1.272	(0.97, 1.67)	0.083
Lead	0.980	(0.81, 1.19)	0.834	.		.
Zinc	1.723	(0.71, 4.19)	0.230	.		.
Iron	0.993	(0.67, 1.46)	0.971	.		.
Aluminum	0.677	(0.48, 0.96)	0.030	.		.
Manganese	0.701	(0.52, 0.95)	0.022	0.560	(0.36, 0.87)	0.010
Cobalt	0.861	(0.65, 1.14)	0.300	.		.
Copper	0.686	(0.37, 1.26)	0.226	.		.
Selenium	0.412	(0.17, 0.99)	0.046	.		.

p = p value, OR = odds ratio, MOR = multivariate odds ratio CI = confidence interval for the odds ratio. Only significant or margninally significant results are shown for multi-variate models.

We hypothesized that the probability of having lung cancer would increase with increasing toenail As concentrations. However, neither the univariate nor the multivariate model found significant increases in the odds of having lung cancer with increasing As concentrations (MOR: 1.03, 95%: CI 0.80–1.34) (**Table 5**). The median As concentration was the same for cases and controls (0.04 μg/g; **Table 4**).

### Impact of dietary variables on trace-element concentrations

Relationship between dietary questions from the 2009–2010 NHANES Dietary Screener and trace element concentrations in toenails are shown in **Table 6**. Within the cases, Al and Fe are significantly positively related to fruit consumption (β = 1.279 & 0.620, respectively); however, they were not significant within controls or the whole cohort. In contrast Co and Pb are positively related to fruit consumption in the whole cohort (β = 0.550 & 0.850, respectively) and Al is negatively related to added sugar intake (β = -0.026), but none were significant within cases or controls (**Table 6**).

**Table 6:** Relationship between dietary variables and select toenail trace-element concentrations.

		All Subjects		Cases		Controls	
Trace Element	Dietary	β (95% CI)	*p*	β (95% CI)	*p*	β (95% CI)	*p*
Al	Pred fruits c/day	0.399 (-0.096–0.895)	0.12	1.279(0.492–2.065)	0.002	-0.435(-1.141–0.271)	0.22
	Pred total added sugars tsp/day	-0.026(-0.052 - -0.001)	0.04	-0.027(-0.067–0.013)	0.18	-0.027(-0.062–0.008)	0.13
Co	Pred fruits c/day	0.550(0.011–1.088)	0.05	0.594(-0.327–1.515)	0.20	0.502(-0.253–1.256)	0.19
Fe	Pred fruits c/day	0.203(-0.193–0.598)	0.31	0.620(0.044–1.196)	0.04	-0.250(-0.846–0.347)	0.41
Pb	Pred fruits c/day	0.850(0.105–1.595)	0.03	1.119(-0.132–2.371)	0.08	0.493(-0.538 - .525)	0.34

The values in the table are β and *p-* values for a multiple regression model that included smoking status, age sex and BMI as co-variates. Models were fit on the whole cohort, cases only and controls only. Only elements and dietary values with p-values that are significant are presented. Pred fruits c/day = predicted fruits in cups (240 mL) per day, tsp = teaspoon (5 mL).

### Drinking water trace-element concentrations

Median method detection limits in drinking water ranged from 0.016 μg/L for Cd and U to 0.54 μg/L for Al. Method detection limits and percentiles broken down by case/control, age and smoking status are presented in **S6 Table.** We found that univariate logistic regression models showed that trace-element concentrations in drinking water were significantly likely to be higher in cancer cases than controls for Co, Al and Cr (**Table 7**); however, only Co remained significant in the multivariate model. However, the concentrations of all elements were very low and quite similar between cases and controls (**S6 Table**; Case medians 0.08, 27 and 0.08 for Co, Al and Cr, respectively).

**Table 7:** Logistic regression results for trace elements in water samples.

	Model					
	Univariate			Multi-variate		
Trace Element	OR	95% CI	p	OR	95% CI	p
Arsenic	1.051	(0.82, 1.34)	0.689	.		
Chromium	1.216	(1.00, 1.47)	0.046	.		
Nickel	1.195	(0.99, 1.45)	0.068	.		
Lead	1.001	(0.87, 1.15)	0.994	.		
Zinc	1.139	(1.00, 1.30)	0.051	.		
Iron	1.091	(0.97, 1.22)	0.138	.		
Aluminum	1.122	(1.02, 1.24)	0.020	.		
Manganese	1.079	(0.97, 1.20)	0.160	.		
Cobalt	1.326	(1.05, 1.68)	0.019	1.463	(1.06, 2.02)	0.021
Copper	0.917	(0.83, 1.02)	0.105	.		
Selenium	0.969	(0.83, 1.14)	0.704	.		

p = p value. Only significant or margninally significant results are shown for multi-variate models.

### Relationship between hair nicotine and Se status

There was no significant overall correlation between hair nicotine and toenail Se concentrations (*r =* -0.09, *p* = 0.114).

### Relationship between toenail and fingernail concentrations

**Fig 1:**
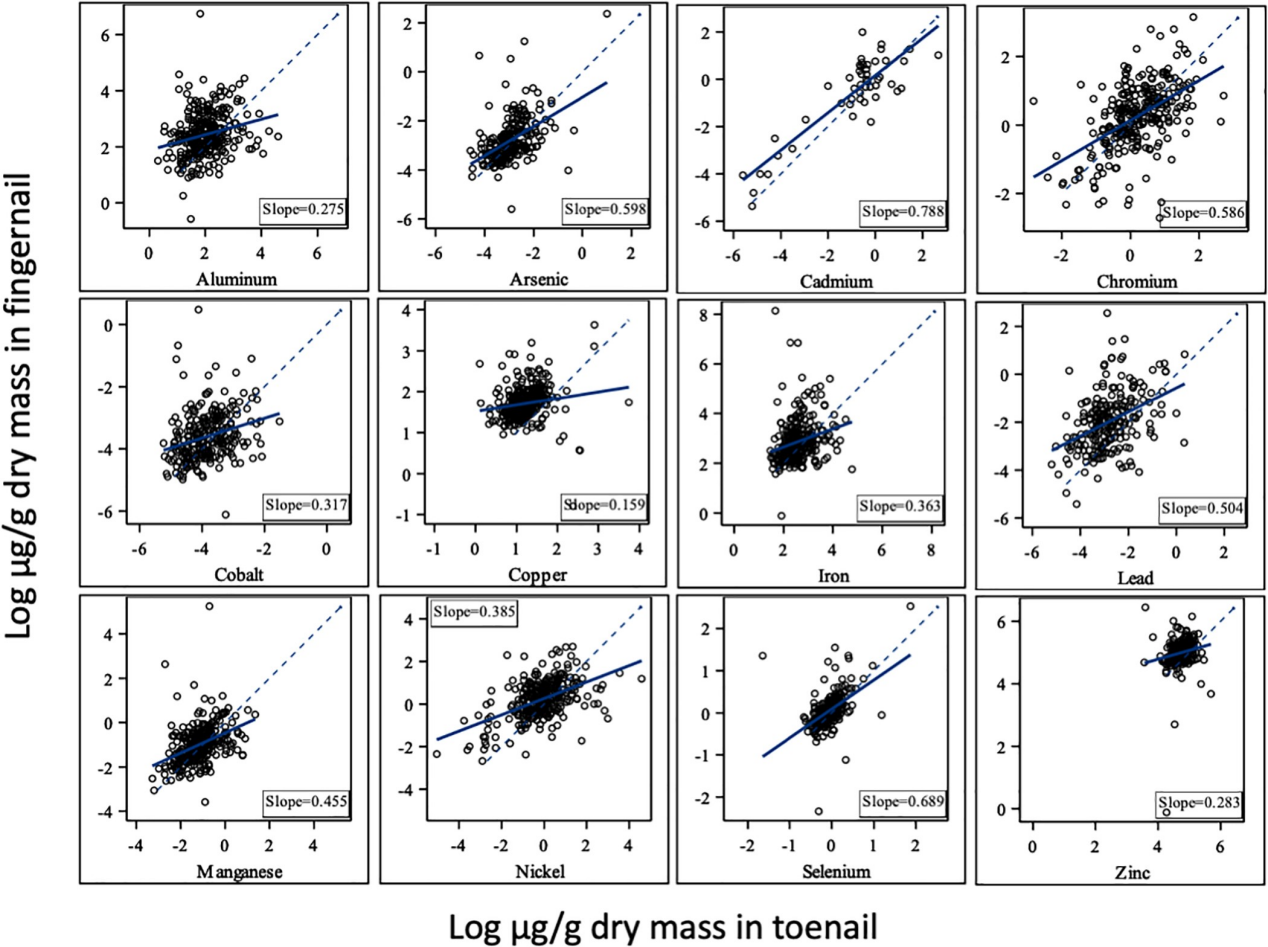
Toenail (x-axis) versus fingernail (y-axis) trace-element concentrations (μg/g). The 1:1 line is dashed and the regression line is solid the slope is given on each panel.

There was not a strong correlation between fingernail and toenail concentrations for any elements (**Fig 1**). Correlation coefficients ranged from 0.15 for Cu to 0.87 for Cd. There was also no consistent slope for the correlations which ranged from 0.159 for Cu to 0.788 for Cd (**Fig 1**.

## Discussion

Based on previous results in an ecologic study from areas with differing lung cancer incidence, we hypothesized that there would be a relationship between lung cancer incidence and toenail arsenic concentrations. Our previous study showed that toenail As concentrations in App KY controls (an area with higher lung cancer incidence) were higher than controls from the metro Louisville area (Jefferson County), an area with lower lung cancer incidence [16]. The median toenail As concentration of App KY residents in our previous study was 0.06 μg/g, similar to 0.04 μg/g in the present study; however, the 90th percentile of concentration was 0.29 μg/g vs 0.13 μg/g in the present study. The distribution in our previous study was skewed having several samples with extremely high (>0.4 μg/g) As concentrations, while urban Jefferson County had a median As concentration 0.02 μg/g and a 90^th^ quintile of 0.04 μg/g. In the present study of App Ky, the overall distribution of As concentrations was similar between cases and controls when comparing concentrations across quintiles, differing by no more than 0.01 μg/g.

Toenail As concentrations in the present study were lower than those in a previous study in a northern App (New Hampshire) population exposed to low/moderate As concentrations in drinking water, which found a positive association between small-cell and squamous-cell carcinoma of the lung when comparing toenail As of > 0.114 μg/g vs < 0.05 μg/g [4]. They also found a positive association between toenail As > 0.05 μg/g combined with history of lung disease and lung carcinomas. Given that our median toenail As concentration fell within their lowest As exposure category, it is not surprising that we found no association with lung cancers. Karagas et al. [25], found that individuals with toenail As concentrations < 0.05 μg/g are generally exposed to drinking water concentrations of < 1 μg/L, well below the United States Environmental Protection Agency (U.S. EPA) maximum contaminant limit (MCL) of 10 μg/L, and consistent with the findings in the present study. In a population exposed to elevated concentrations of As in soil (median of 92 mg/kg) and drinking water (median of 43.8 μg/L), toenail concentrations were found to be 32.1 and 21.7 μg/g, respectively [26]. Therefore, our data indicate that, by and large, individuals examined in the current study did not have recent elevated exposure to As.

Several trace-elements which can either be micro-nutrients or toxicants showed decreased odds of having lung cancer with increasing toenail concentrations. For example, when examined using univariate logistic regression, probability of having lung cancer decreased with increasing Se concentrations (OR: 0.41, CI: 0.17–0.99). However, this did not hold in multivariate analyses possibly due to confounding factors. A previous study suggested that the inverse relationship between Se status and bladder cancer was confounded by smoking [27]. However, a study relating Se concentration to pancreatic cancer found a significant decrease in cancer risk with increasing Se concentrations even after controlling for smoking [28]. Cases and controls in the present study who were former smokers had similar, although significantly different (*p =* 0.04) median toenail Se concentrations (0.90 and 0.97 μg/g respectively). Likewise, cases and controls who were smokers had median toenail Se concentrations which were not significantly different (0.87 vs 0.86 μg/g; *p* = 0.89) as did never smokers (1.06 vs 0.97 μg/g; *p* = 0.25). When just comparing cases vs controls regardless of smoking status, cases had a statistically significant decrease in Se concentrations (median 0.90 vs 0.94 μg/g; *p* = 0.01). However, given the small difference between cases and controls relevant to smoking status, it is not surprising that Se concentrations were not significantly related to lung cancer status when pack years was included in the multi-variate logistic regression model. Because prior studies had shown that Se status could be lowered by smoking we used hair nicotine as an independent confirmation of smoking status. There was no significant relationship between hair nicotine and toenail Se concentrations. It is therefore likely that there is some other proximate cause of the lower Se concentrations in smokers. Other studies have shown that differences in supplement use account for lower Se status in smokers [29]; however, we did not collect vitamin supplement use data and cannot test this hypothesis.

We also hypothesized that Cr would be elevated in cases relative to controls based on our previous study, where we found that Cr concentrations in App Ky controls were 0.27 μg/g versus 0.04 μg/g in Jefferson County controls. In the present study, we found that Cr concentrations were inversely associated with lung cancer in the univariate logistic model (OR 0.70; CI 0.55–0.89). The median Cr concentrations were 0.93 μg/g in cases and 1.17μg/g in controls. These concentrations are similar to what has been observed in control groups in other studies. For example, in a prospective case-control study of breast cancer in the U.S. Nurses’ Health Study cohort, cases and controls had geometric mean concentrations of 1.16 and 1.13 μg Cr/g, respectively [30]. There was one study where controls from a population in Spain had lower concentrations (median = 0.40 μg/g and mean = 1.11μg/g) [28]. Lower concentrations of Cr can be related to deficiency. While the role of Cr as a toxicant/carcinogen has been explored for lung cancer, its role as a nutrient in relation to lung cancer has not been explored to our knowledge. Cr deficiency has been associated with several other diseases. The App Ky population had toenail Cr concentrations similar to those that have been associated with increased risk of non-fatal myocardial infarction in men in a previous study where risk decreased with increasing Cr concentrations [31]. In that study cases had mean Cr concentrations of 1.10 μg/g and controls had mean Cr concentrations of 1.30 μg/g. Female never smokers and former smokers (1.40 and 1.31 μg/g, respectively) and female controls (1.34 μg/g) from our study had geometric mean toenail Cr concentrations exceeding the mean concentrations associated with myocardial infarction in Rajpathak et al., [32]. Female never smoker controls had geometric mean toenail concentrations of 1.42 μg Cr/g. We also found that females had higher median toenail Cr concentrations than males regardless of case/control status (1.22 vs 0.98 μg/g, p = 0.007). The pattern of lower Cr concentrations in cancer cases, men, and smokers, may be related to the fact that trivalent Cr is an essential nutrient derived from the diet or supplements. It is not carcinogenic as opposed to hexavalent chromium [33]. Cancer cases may have had poorer Cr nutrition relative to controls. Baseline Cr in concentrations in nails are likely derived primarily from trivalent Cr [31]. Although the biological function of trivalent chromium is poorly understood, it plays a role in carbohydrate, fat and protein metabolism [31]. It is also known to enhance the action of insulin and insulin resistance has been associated with Cr deficiency [34]. Southeastern Kentucky has type II diabetes prevalence (~18%) that exceeds the state as a whole (~13.4%) and far exceeds the national rate (~6%) [35].

Manganese concentrations were lower in cancer cases than controls in both multivariate and univariate models (MOR = 0.56; CI = 0.36–0.87). Decreased Mn status has previously been associated with laryngeal cancer [36]. Conversely, increased Mn concentrations have been associated with prostate cancer [37]. Manganese is also a critical component of antioxidant defenses through Mn-superoxide dismutase [38, 39]. This is the first study we are aware of to find an inverse relationship between Mn concentrations in toenails and lung cancer status.

While Ni had no significant association with lung cancer risk, our previous study did suggest higher values in App Ky than Jefferson County [16] and Ni is likely a lung carcinogen when exposure occurs by inhalation [40]. The present study showed no significant relationship with cancer incidence (multivariate, p = 0.08), but higher values than in our previous study [16]. Amaral and colleagues [28] found that Ni concentrations in toenails were significantly higher in controls than pancreatic cancer cases (median = 0.39 vs 0.23 μg /g, respectively). While our cases and controls had median values that were higher than this (0.70 and 0.89 μg /g, respectively), they were still lower than values found in mining and non-mining communities in Zambia (geometric mean = 1.99 and 1.21 μg /g, respectively) [41]. They were also lower than toenail Ni concentration in welders (median = 2.19 μg /g) [42] and similar to Ni concentrations found in unexposed volunteers (mean = 0.78 μg /g) [43].

Our study was not specifically designed to elucidate the relationship between diet and trace-element concentrations, thus the dietary questions in our questionnaire only asked about broad categories of dietary intake, such fruit and vegetable intake, and thus lack sufficient specificity to establish relationships between particular dietary choices and trace-element intake. Also, a previous study found that dietary parameters are less correlated with toenail trace-element concentrations as compared to correlations between trace element concentrations in water and toenails [44]. Specifically for As, arsenobetaine, which is abundant in seafood, is less likely to be incorporated into toenails than the more toxic inorganic forms due to its lack of metabolism and rapid elimination in the urine [45]. Shellfish intake is better correlated with toenail As than fish, likely due to the greater proportion of inorganic arsenic found in shellfish, but accounts for less of the variation in toenail As than drinking water [46]. This is particularly true in the United States where overall fish consumption is moderate [45].

Another potential source of trace-element exposure is drinking water. Only a few individual drinking water samples were over ½ of the U.S. EPA maximum contaminant level. Of these the majority of cases were for Pb and Cu, with only one case where Cd exceeded this level. U.S. EPA drinking water standards for Cr and Al are 100, and 25,000 μg/L. Given that these concentrations are so low, drinking water likely served as a very minor component of trace-element intake. For example, for Co, the average daily intake is about 11 μg/day [47] versus the 0.16 μg that would result from ingesting 2 L of drinking water per day at 0.08 μg/L. This explains why the differences in drinking water trace-element concentrations are not reflected in toenail trace-element concentrations. The significance of these findings are not clear given the low concentrations in water, and warrants further investigation.

Toenails, rather than fingernails, are accepted as a non-invasive biomarker of trace-element exposure in human subjects; toenails are less exposed to external contamination and have a slower rate of growth (approximately half the rate of fingernails), so they integrate a longer exposure interval per unit length [15]. However, for many subjects, it may not be possible to obtain toenails because of amputations or because of the risk of infection in subjects with diabetes, raising the potential for fingernails as a surrogate of toenails. Unfortunately, our results indicate that fingernail and toenail concentrations are poorly correlated. Poor correlations could be due to differences in integration time due to differential growth of the two nail types, differential external contamination, differences in blood supply between hands and feet, or a combination of these factors. As the largest study to date comparing fingernail to toenail concentrations, we conclude that fingernail concentrations are not a useful surrogate for toenail concentrations.

This population-based, case-control study has a few limitations. There were a few slight differences in the characteristics of the case and control population. Controls had higher median BMI (28.7 vs 26.7), a higher level of education, and a higher median household income. As expected, the cases had a significantly higher percentage of smokers compared to controls. There were also differences between subjects that provided nail samples versus those who didn’t. The population of participants that provided nail samples was younger (median age 62 vs 65), had a significantly higher percentage of females (56.9 vs 47.7), a significantly higher level of education and a significantly higher median household income. Additionally, the recruitment rates for the study were relatively low, perhaps due to several factors, such as the health status of the cases, reliance on dialing phone numbers to recruit controls, and the need for a home visit. The individuals who were recruited tended to be slightly older than the individuals contacted and more females than males were recruited. These slight differences in the case and control populations and the recruited versus contacted populations have the potential to introduce bias in the trace-element concentrations due to different patterns of exposure due to demographic status. To address these limitations, we used multivariate logistic regression models to control for confounding variables which were different between the case/control, recruited/contacted and provided nail/didn’t provide nail populations. We found only BMI and smoking status to be significant co-variates for trace-element concentrations and included them in our multi-variate logistic regression models. Finally, it should also be noted that toenail analyses integrate at best a few months of exposure, while lung cancer takes potentially decades to develop.

## Conclusions

Our case-control study provides meaningful participant-level assessment of a randomly selected controls from an area of highest incidence of lung cancer in the United States compared to cancer patients from the same region. Contrary to our initial hypothesis, based on previous studies, we found that there was no positive association between trace-element concentrations, in toenails and lung cancer in our study population. We found a negative association between toenail concentrations and lung cancers for several elements in univariate models (Al, Mn, Cr, and Se) with only Mn being significant in multivariate analysis. Overall, toenail trace element concentrations were similar to or lower than those of controls from a variety of previously studied populations, perhaps accounting for the observed lack of positive correlation with lung cancer incidence. For some elements, such as for Se, Cr and Mn, negative associations with lung cancer may point to nutritional differences in the population that could be associated with cancers or other chronic health conditions; however, a lack of significance in our multivariate models indicates that confounding factors likely explain these associations. While some significant differences in drinking water concentrations were found between cases and controls, the concentrations of trace elements in drinking water were low and were not reflected in toenail concentrations. Additionally, we found that fingernails are not a reliable surrogate for assessing mid-term trace element exposure.

## Supporting information

S1 Table Full cohort demographics broken down by case versus control. RTA = Refused to answer, DK = didn’t know, pred = predicted, mos = months, vegs = vegetables, inc = including, c/day = cups (240 mL) per day, tsp = teaspoon (5 mL), bevs–beverages, gr = grade, educ = education, K = thousand, COPD = chronic obstructive pulmonary disease.
(PDF)

S2 Table Full cohort demographics broken down by those who provided toenails (nails) versus those who didn’t (no-nails). RTA = Refused to answer, DK = didn’t know, pred = predicted, mos = months, vegs = vegetables, inc = including, c/day = cups (240 mL) per day, tsp = teaspoon (5 mL), bevs–beverages, gr = grade, educ = education, K = thousand, COPD = chronic obstructive pulmonary disease. Numbers in parenthesis indicate the range of values for age and BMI and percentages for count data.
(PDF)

S3 Table Number of individuals contacted versus recruited by gender and age for controls (top) and cases (bottom).
(PDF)

S4 Table Recovery of trace elements from National Institute of Environmental Sciences (Japan) certified reference material number 13 (Human Hair). Values (μg/g dry mass) are means followed by standard deviation or uncertainty in parenthesis for measured and certified or reference values, respectively. Asterisks indicate that the value is a reference value with no uncertainty estimate.
(PDF)

S5 Table Quintiles of trace-element concentrations in toenails (μg/g dry mass) broken down by case versus control, age and smoking status. (PDF)

S6 Table Quintiles of drinking water trace element concentrations and method detection limits. (μg/L).
(PDF)
