# Supplementary material for: A case-control study of trace-element status and lung cancer in Appalachian Kentucky
Source: PLoS One. 2019 Feb 27;14(2):e0212340. doi: 10.1371/journal.pone.0212340 (PMC6392268; doi:10.1371/journal.pone.0212340)
Supplement: S1 Table — RTA = Refused to answer, DK = didn’t know, pred = predicted, mos = months, vegs = vegetables, inc = including, c/day = cups (240 mL) per day, tsp = teaspoon (5 mL), bevs–beverages, gr = grade, educ = education, K = thousand, COPD = chronic obstructive pulmonary disease. (PDF) [file pone.0212340.s001.pdf]

S1 Table. Full cohort demographics broken down by case versus control.

		Case	Control	All	p-value
N		150	370	520	
Age		62.5 (39.0 - 89.0)	63.0 (26.0 - 91.0)	63.0 (26.0 - 91.0)	0.62
BMI (3 mos ago)		26.4 (14.6 - 55.2)	29.0 (15.3 - 66.6)	28.4 (14.6 - 66.6)	<0.0001
Created: Comorbidity Score		2.0 (0.0 - 11.0)	1.0 (0.0 - 11.0)	1.0 (0.0 - 11.0)	<0.0001
Pred fruits/vegs incl legumes & fries c/day		2.4 (1.6 - 3.7)	2.6 (1.6 - 4.2)	2.5 (1.6 - 4.2)	0.01
Pred fruits/vegs incl legumes & no fries c/day		2.3 (1.4 - 3.5)	2.4 (1.4 - 4.1)	2.4 (1.4 - 4.1)	0.02
Pred fruits c/day		0.8 (0.4 - 2.2)	0.9 (0.4 - 2.0)	0.9 (0.4 - 2.2)	0.21
Pred vegetables incl legumes & fries c/day		1.6 (1.1 - 2.6)	1.7 (1.1 - 3.1)	1.6 (1.1 - 3.1)	0.03
Pred vegs incl legumes & no fries c/day		1.5 (1.0 - 2.5)	1.6 (0.9 - 3.0)	1.5 (0.9 - 3.0)	0.03
Pred dairy c/day		1.5 (0.9 - 3.4)	1.5 (0.9 - 4.3)	1.5 (0.9 - 4.3)	0.82
Pred total added sugars tsp/day		15.3 (10.0 - 36.0)	15.0 (10.0 - 36.8)	15.1 (10.0 - 36.8)	0.26
Pred added sugars from sugar-sweetened bevs tsp/da		6.2 (3.6 - 21.9)	5.1 (3.6 - 20.9)	5.3 (3.6 - 21.9)	0.03
Pred whole grains oz/day		0.6 (0.3 - 1.8)	0.8 (0.3 - 1.9)	0.8 (0.3 - 1.9)	0.02
Pred fiber m/day		15.9 (11.1 - 25.5)	16.9 (10.0 - 28.5)	16.6 (10.0 - 28.5)	0.005
Pred calcium mg/day		900.9 (632.2 - 1483.9)	931.0 (615.8 - 1797.5)	921.3 (615.8 - 1797.5)	0.09
Gender	Female	88 (58.7%)	194 (52.4%)	282 (54.2%)	0.20
	Male	62 (41.3%)	176 (47.6%)	238 (45.8%)	
Race	White	147 (98.0%)	365 (98.6%)	512 (98.5%)	0.70
	Non-White	3 (2.0%)	5 (1.4%)	8 (1.5%)	
Education		1 (0.7%)	1 (0.3%)	2 (0.4%)	<0.0001
	8th Gr or Less	36 (24.0%)	31 (8.4%)	67 (12.9%)	
	Grade 9-11	26 (17.3%)	25 (6.8%)	51 (9.8%)	
	HS Educ	48 (32.0%)	138 (37.3%)	186 (35.8%)	
	College Educ	39 (26.0%)	175 (47.3%)	214 (41.2%)	
Household Income		2 (1.3%)	0 (0.0%)	2 (0.4%)	<0.0001
	<$15K	54 (36.0%)	51 (13.8%)	105 (20.2%)	
	$15K-<$25K	37 (24.7%)	58 (15.7%)	95 (18.3%)	
	$25K-<$50K	24 (16.0%)	99 (26.8%)	123 (23.7%)	

	$50K-<$75K	5 (3.3%)	54 (14.6%)	59 (11.3%)	
	$75K +	7 (4.7%)	56 (15.1%)	63 (12.1%)	
	RTA/DK	21 (14.0%)	52 (14.1%)	73 (14.0%)	
Smoker	Current	77 (51.3%)	67 (18.1%)	144 (27.7%)	<0.0001
	Former	67 (44.7%)	118 (31.9%)	185 (35.6%)	
	Never	6 (4.0%)	185 (50.0%)	191 (36.7%)	
Diabetes	No	117 (78.0%)	303 (81.9%)	420 (80.8%)	0.31
	Yes	33 (22.0%)	67 (18.1%)	100 (19.2%)	
COPD	No	83 (55.3%)	329 (88.9%)	412 (79.2%)	<0.0001
	Yes	65 (43.3%)	39 (10.5%)	104 (20.0%)	
	D	2 (1.3%)	2 (0.5%)	4 (0.8%)	
Chronic Lung Disease	No	56 (37.3%)	256 (69.2%)	312 (60.0%)	<0.0001
	Yes	94 (62.7%)	114 (30.8%)	208 (40.0%)	
Chronic Heart Disease	No	100 (66.7%)	279 (75.4%)	379 (72.9%)	0.04
	Yes	50 (33.3%)	91 (24.6%)	141 (27.1%)	

RTA= Refused to answer, DK = didn’t know, pred = predicted, mos = months, vegs = vegetables, inc = including, c/day= cups (240 mL) per day, tsp = teaspoon (5 mL), bevs – beverages, gr = grade, educ = education, K = thousand, COPD = chronic obstructive pulmonary disease.
