# Supplementary material for: A case-control study of trace-element status and lung cancer in Appalachian Kentucky
Source: PLoS One. 2019 Feb 27;14(2):e0212340. doi: 10.1371/journal.pone.0212340 (PMC6392268; doi:10.1371/journal.pone.0212340)
Supplement: S2 Table — RTA = Refused to answer, DK = didn’t know, pred = predicted, mos = months, vegs = vegetables, inc = including, c/day = cups (240 mL) per day, tsp = teaspoon (5 mL), bevs–beverages, gr = grade, educ = education, K = thousand, COPD = chronic obstructive pulmonary disease. Numbers in parenthesis indicate the range of values for age and BMI and percentages for count data. (PDF) [file pone.0212340.s002.pdf]

S2 Table. Full cohort demographics broken down by those who provided toenails (nails) versus those who didn't (no-nails).

		Nails	No Nails	All	p-value
N		367	153	520	
Age		62.0 (26.0 - 91.0)	65.0 (38.0 - 87.0)	63.0 (26.0 - 91.0)	0.01
BMI (3 mos ago)		28.5 (14.6 - 66.6)	28.2 (15.1 - 47.3)	28.4 (14.6 - 66.6)	0.83
Created: Comorbidity Score		1.0 (0.0 - 11.0)	1.0 (0.0 - 11.0)	1.0 (0.0 - 11.0)	0.62
Pred fruits/vegs incl legumes & fries c/day		2.5 (1.6 - 4.2)	2.5 (1.6 - 4.2)	2.5 (1.6 - 4.2)	0.69
Pred fruits/vegs incl legumes & no fries c/day		2.4 (1.4 - 4.1)	2.4 (1.5 - 4.0)	2.4 (1.4 - 4.1)	0.62
Pred fruits c/day		0.8 (0.4 - 2.2)	0.9 (0.4 - 1.9)	0.9 (0.4 - 2.2)	1.00
Pred vegetables incl legumes & fries c/day		1.7 (1.1 - 2.7)	1.6 (1.2 - 3.1)	1.6 (1.1 - 3.1)	0.34
Pred vegs incl legumes & no fries c/day		1.5 (0.9 - 2.5)	1.5 (1.0 - 3.0)	1.5 (0.9 - 3.0)	0.21
Pred dairy c/day		1.5 (0.9 - 4.3)	1.5 (0.9 - 2.7)	1.5 (0.9 - 4.3)	0.16
Pred total added sugars tsp/day		14.9 (10.0 - 36.0)	15.4 (10.0 - 36.8)	15.1 (10.0 - 36.8)	0.11
Pred added sugars from sugar-sweetened bevs tsp/da		5.6 (3.6 - 21.9)	5.1 (3.6 - 20.9)	5.3 (3.6 - 21.9)	0.94
Pred whole grains oz/day		0.8 (0.3 - 1.8)	0.7 (0.3 - 1.9)	0.8 (0.3 - 1.9)	0.26
Pred fiber gm/day		16.4 (11.1 - 25.5)	17.2 (10.0 - 28.5)	16.6 (10.0 - 28.5)	0.15
Pred calcium mg/day		906.6 (632.2 - 1797.5)	948.0 (615.8 - 1477.5)	921.3 (615.8 - 1797.5)	0.01
Case/Control	case	100 (27.2%)	50 (32.7%)	150 (28.8%)	0.21
	control	267 (72.8%)	103 (67.3%)	370 (71.2%)	
Gender	Female	209 (56.9%)	73 (47.7%)	282 (54.2%)	0.05
	Male	158 (43.1%)	80 (52.3%)	238 (45.8%)	
Race	White	360 (98.1%)	152 (99.3%)	512 (98.5%)	0.45
	Non-White	7 (1.9%)	1 (0.7%)	8 (1.5%)	
Education	.	1 (0.3%)	1 (0.7%)	2 (0.4%)	0.03
	8th Gr or Less	39 (10.6%)	28 (18.3%)	67 (12.9%)	
	Grade 9-11	36 (9.8%)	15 (9.8%)	51 (9.8%)	
	HS Educ	127 (34.6%)	59 (38.6%)	186 (35.8%)	
	College Educ	164 (44.7%)	50 (32.7%)	214 (41.2%)	
Household Income	.	2 (0.5%)	0 (0.0%)	2 (0.4%)	0.0002
	<$15K	72 (19.6%)	33 (21.6%)	105 (20.2%)	

	$15K-<$25K	70 (19.1%)	25 (16.3%)	95 (18.3%)	
	$25K-<$50K	91 (24.8%)	32 (20.9%)	123 (23.7%)	
	$50K-<$75K	46 (12.5%)	13 (8.5%)	59 (11.3%)	
	$75K +	51 (13.9%)	12 (7.8%)	63 (12.1%)	
	RTA/DK	35 (9.5%)	38 (24.8%)	73 (14.0%)	
		Nails	No Nails	All	p-value
Smoker	Current	102 (27.8%)	42 (27.5%)	144 (27.7%)	0.35
	Former	124 (33.8%)	61 (39.9%)	185 (35.6%)	
	Never	141 (38.4%)	50 (32.7%)	191 (36.7%)	
Diabetes	No	303 (82.6%)	117 (76.5%)	420 (80.8%)	0.11
	Yes	64 (17.4%)	36 (23.5%)	100 (19.2%)	
COPD	No	288 (78.5%)	124 (81.0%)	412 (79.2%)	0.41
	Yes	77 (21.0%)	27 (17.6%)	104 (20.0%)	
	D	2 (0.5%)	2 (1.3%)	4 (0.8%)	
Chronic Lung Disease	No	214 (58.3%)	98 (64.1%)	312 (60.0%)	0.22
	Yes	153 (41.7%)	55 (35.9%)	208 (40.0%)	
Chronic Heart Disease	No	273 (74.4%)	106 (69.3%)	379 (72.9%)	0.23
	Yes	94 (25.6%)	47 (30.7%)	141 (27.1%)	

RTA= Refused to answer, DK = didn't know, pred = predicted, mos = months, vegs = vegetables, inc = including, c/day= cups (240 mL) per day, tsp = teaspoon (5 mL), bevs – beverages, gr = grade, educ = education, K = thousand, COPD = chronic obstructive pulmonary disease. Numbers in parenthesis indicate the range of values for age and BMI and percentages for count data.
