# Supplementary material for: A case-control study of trace-element status and lung cancer in Appalachian Kentucky
Source: PLoS One. 2019 Feb 27;14(2):e0212340. doi: 10.1371/journal.pone.0212340 (PMC6392268; doi:10.1371/journal.pone.0212340)
Supplement: S3 Table — Number of individuals contacted versus recruited by gender and age for controls (top) and cases (bottom). (PDF) [file pone.0212340.s003.pdf]

S3 Table. Number of individuals contacted versus recruited by gender and age for controls (top) and cases (bottom).

Control

Variable	Total			Male			Female			
	Sample	Yes for to be recruited	Yes recruited in the study	Sample	Yes for to be recruited	Yes recruited in the study	Sample	Yes for to be recruited	Yes recruited in the study	
	N	N	N	N	N	N	N	N	N	
Age Group										
18 - 34	55	8	1	20	3	1	35	5		
35-49	2160	280	9	1087	143	0	1073	137	9	
50-64	4373	845	124	2485	431	62	1888	414	62	
65-74	2558	669	138	1291	323	59	1267	346	79	
75-84	1435	271	81	667	116	45	768	155	36	
85+	332	42	17	132	14	9	200	28	8	
NULL	2	0	0	1	0	0	0	0	0	
	10,915	2,115	370	5,683	1,030	176	5,231	1,085	194	370 total controls

Case

Variable	Total			Male			Female				
	Sample	Yes for to be recruited	Yes recruited in the study	Sample	Yes for to be recruited	Yes recruited in the study	Sample	Yes for to be recruited	Yes recruited in the study		
	N	N	N	N	N	N	N	N	N		
Age Group											
18 - 34	43	0	0	16	0	0	27	0	0		
35-49	248	26	6	90	12	2	158	14	4		
50-64	1048	277	54	549	116	21	499	161	33		
65-74	911	213	56	480	109	24	431	104	32		
75-84	435	133	27	254	70	10	181	63	17		
85+	69	116	7	36	6	5	33	7	2		
NULL	22	5	0	13	3	0	9	2	0		
Stage											
Stage I											
Stage II											
Stage III											
Stage IV	2,776	770	150	1,438	316	62	1,338	351	88	150	total cases
