# Supplementary material for: A case-control study of trace-element status and lung cancer in Appalachian Kentucky
Source: PLoS One. 2019 Feb 27;14(2):e0212340. doi: 10.1371/journal.pone.0212340 (PMC6392268; doi:10.1371/journal.pone.0212340)
Supplement: S4 Table — Values (μg/g dry mass) are means followed by standard deviation or uncertainty in parenthesis for measured and certified or reference values, respectively. Asterisks indicate that the value is a reference value with no uncertainty estimate. (PDF) [file pone.0212340.s004.pdf]

S4 Table. Recovery of trace elements from National Institute of Environmental Sciences (Japan) certified reference material number 13 (Human Hair).

	**Measured (CRM 13)**	**Certified (CRM 13)**
**Al**	58.8 (12.7)	120*
**Cr**	1.27 (0.45)	NA
**Mn**	2.89 (0.35)	3.9*
**Fe**	99.7 (17.5)	140*
**Co**	0.073 (0.015)	0.07*
**Ni**	1.69 (0.36)	NA
**Cu**	15.4 (1.19)	15.3 (1.3)
**Zn**	157.9 (10.7)	172 (11)
**As**	0.094 (0.016)	0.10*
**Se**	1.73 (0.12)	1.79 (0.17)
**Cd**	0.204 (0.017)	0.23 (0.03)
**Pb**	4.78 (0.61)	4.6 (0.4)
**U**	BDL	NA

Values (μg/g dry mass) are means followed by standard deviation or uncertainty in parenthesis for measured and certified or reference values, respectively. Asterisks indicate that the value is a reference value with no uncertainty estimate.
