# Supplementary material for: A case-control study of trace-element status and lung cancer in Appalachian Kentucky
Source: PLoS One. 2019 Feb 27;14(2):e0212340. doi: 10.1371/journal.pone.0212340 (PMC6392268; doi:10.1371/journal.pone.0212340)
Supplement: S5 Table — (PDF) [file pone.0212340.s005.pdf]

S5 Table – Quintiles of trace-element concentrations in toenails (μg/g dry mass) broken down by case versus control, age and smoking status.

		Case/Control		Age				Smoking Status					
				<63		63+		Current		Former		Never	
		case	control	case	control	case	control	case	control	case	control	case	control
As	10th	0.01	0.01	0.02	0.02	0.01	0.01	0.02	0.02	0.01	0.01	0.01	0.01
	25th	0.03	0.02	0.03	0.03	0.03	0.02	0.03	0.03	0.03	0.02	0.01	0.02
	50th	0.04	0.04	0.05	0.05	0.03	0.04	0.05	0.05	0.04	0.04	0.04	0.04
	75th	0.06	0.07	0.09	0.08	0.05	0.06	0.07	0.10	0.06	0.06	0.04	0.07
	90th	0.13	0.12	0.14	0.13	0.10	0.10	0.15	0.13	0.11	0.10	0.04	0.11
Cr	10th	0.22	0.35	0.29	0.34	0.15	0.37	0.21	0.23	0.24	0.42	0.68	0.34
	25th	0.51	0.71	0.54	0.73	0.45	0.68	0.45	0.65	0.54	0.71	0.68	0.74
	50th	0.93	1.17	0.92	1.13	0.98	1.21	0.77	0.95	1.04	1.12	1.02	1.37
	75th	1.60	2.28	1.23	1.91	1.72	2.69	1.25	1.65	1.72	2.14	1.29	2.62
	90th	2.29	3.80	2.21	3.05	2.38	3.85	2.23	2.06	2.50	3.35	1.29	4.39
Ni	10th	0.16	0.11	0.27	0.12	0.07	0.11	0.23	0.11	0.10	0.14	0.43	0.08
	25th	0.38	0.43	0.40	0.42	0.36	0.45	0.39	0.38	0.36	0.42	0.43	0.52
	50th	0.70	0.89	0.69	0.79	0.71	0.96	0.57	0.62	0.77	0.95	0.82	1.03
	75th	1.19	1.78	1.23	1.63	1.18	1.83	1.23	1.07	1.21	1.82	1.16	1.87
	90th	2.37	3.01	2.47	2.48	2.03	3.43	1.89	1.84	3.90	3.01	1.16	3.43
Cd	10th	0.00	0.01	0.00	0.01	0.00	0.01	0.00	0.01	0.00	0.00	0.00	0.01
	25th	0.01	0.01	0.00	0.01	0.01	0.01	0.01	0.01	0.01	0.01	0.00	0.02
	50th	0.02	0.02	0.02	0.02	0.02	0.03	0.02	0.02	0.02	0.02	0.02	0.03
	75th	0.05	0.07	0.04	0.07	0.07	0.07	0.05	0.05	0.06	0.07	0.03	0.08
	90th	0.61	0.52	0.43	0.35	0.69	0.53	0.54	0.14	0.69	0.54	0.03	0.52
Pb	10th	0.01	0.01	0.01	0.01	0.00	0.01	0.01	0.02	0.01	0.01	0.00	0.01
	25th	0.02	0.02	0.02	0.02	0.01	0.02	0.02	0.03	0.01	0.02	0.00	0.01
	50th	0.04	0.04	0.05	0.04	0.03	0.04	0.04	0.07	0.03	0.05	0.01	0.03
	75th	0.08	0.09	0.10	0.09	0.08	0.09	0.07	0.14	0.12	0.10	0.08	0.07
	90th	0.20	0.18	0.16	0.15	0.32	0.23	0.15	0.19	0.32	0.22	0.08	0.16

		Case/Control		Age				Smoking Status					
				<63		63+		Current		Former		Never	
		case	control	case	control	case	control	case	control	case	control	case	control
Zi	10th	80.29	84.19	87.64	84.88	79.28	83.77	82.12	82.49	77.56	83.77	87.86	85.23
	25th	96.91	92.90	99.42	93.76	95.10	92.61	98.60	93.76	96.50	91.95	87.86	93.43
	50th	116.92	109.18	117.92	108.49	115.93	110.73	117.92	108.59	116.93	109.29	89.35	109.23
	75th	139.33	130.41	142.92	129.80	130.79	130.56	140.03	122.37	138.75	132.65	144.55	130.43
	90th	162.76	147.09	163.66	144.48	160.25	147.94	162.91	144.48	160.25	149.14	144.55	146.77
U	10th	0.00	0.00	0.00	0.00	0.00	0.00	0.00	0.00	0.00	0.00	0.00	0.00
	25th	0.00	0.00	0.00	0.00	0.00	0.00	0.00	0.00	0.00	0.00	0.00	0.00
	50th	0.00	0.00	0.00	0.00	0.00	0.00	0.00	0.00	0.00	0.00	0.00	0.00
	75th	0.01	0.01	0.01	0.01	0.01	0.01	0.00	0.00	0.01	0.01	0.01	0.01
	90th	0.01	0.01	0.01	0.01	0.01	0.01	0.01	0.01	0.02	0.01	0.01	0.01
Fe	10th	6.32	6.52	9.01	6.99	5.85	5.71	7.71	8.06	5.85	6.41	8.14	6.30
	25th	9.43	8.65	9.89	9.21	8.14	7.84	9.67	11.13	9.19	8.01	8.14	8.12
	50th	11.79	12.03	12.76	12.21	11.27	11.73	11.67	13.32	11.35	12.03	39.15	10.86
	75th	15.68	17.12	18.77	16.73	14.03	17.43	15.04	21.51	15.22	17.74	39.30	15.93
	90th	29.13	26.82	30.67	26.74	26.58	27.91	22.34	34.72	30.78	29.34	39.30	23.31
Al	10th	2.83	3.68	3.25	4.14	2.05	3.58	3.10	3.58	2.83	4.03	2.05	3.33
	25th	4.16	5.17	5.20	5.62	3.35	4.60	4.09	5.31	4.44	4.78	2.05	5.35
	50th	6.38	7.30	7.64	8.09	5.82	6.41	6.44	7.95	6.24	7.45	7.96	7.23
	75th	9.14	11.40	10.12	11.85	7.46	11.12	8.64	12.04	10.12	12.87	9.54	10.18
	90th	13.77	18.67	16.00	22.97	11.32	14.98	11.32	18.67	16.62	24.02	9.54	15.27
Mn	10th	0.10	0.13	0.11	0.13	0.09	0.11	0.11	0.13	0.09	0.12	0.11	0.13
	25th	0.16	0.20	0.23	0.21	0.13	0.18	0.18	0.19	0.14	0.23	0.11	0.20
	50th	0.27	0.32	0.31	0.31	0.24	0.33	0.28	0.31	0.27	0.34	0.32	0.31
	75th	0.41	0.51	0.44	0.49	0.34	0.51	0.44	0.52	0.37	0.54	0.40	0.48
	90th	0.63	0.92	0.67	1.01	0.51	0.86	0.54	0.93	0.73	1.01	0.40	0.76
Co	10th	0.01	0.01	0.01	0.01	0.01	0.01	0.01	0.01	0.01	0.01	0.00	0.01
	25th	0.01	0.01	0.01	0.01	0.01	0.01	0.01	0.01	0.01	0.01	0.00	0.01
	50th	0.02	0.02	0.02	0.02	0.02	0.02	0.01	0.02	0.02	0.02	0.01	0.02
	75th	0.02	0.03	0.02	0.03	0.02	0.04	0.02	0.03	0.03	0.03	0.02	0.03
	90th	0.05	0.05	0.04	0.05	0.05	0.05	0.03	0.04	0.06	0.05	0.02	0.05

		Case/Control		Age				Smoking Status					
				<63		63+		Current		Former		Never	
		case	control	case	control	case	control	case	control	case	control	case	control
**Cu**	**10th**	2.13	2.24	2.18	2.37	2.01	2.00	2.18	1.92	2.01	2.06	2.63	2.29
	**25th**	2.57	2.72	2.61	2.84	2.55	2.61	2.58	2.46	2.49	2.67	2.63	2.81
	**50th**	3.09	3.25	3.11	3.46	3.02	3.08	3.01	3.09	3.16	3.38	3.02	3.25
	**75th**	4.00	4.11	4.02	4.20	3.98	4.00	3.75	3.93	4.12	4.15	4.63	4.12
	**90th**	5.01	5.06	4.83	5.40	5.08	4.75	4.74	6.00	5.24	5.18	4.63	5.01
**Se**	**10th**	0.67	0.74	0.68	0.74	0.67	0.73	0.68	0.69	0.67	0.74	0.94	0.75
	**25th**	0.74	0.84	0.72	0.85	0.76	0.83	0.74	0.78	0.74	0.85	0.94	0.85
	**50th**	0.90	0.94	0.86	0.93	0.91	0.95	0.87	0.86	0.90	0.97	1.06	0.97
	**75th**	1.05	1.08	1.03	1.05	1.05	1.08	1.02	0.97	1.05	1.08	1.40	1.10
	**90th**	1.17	1.26	1.18	1.22	1.16	1.27	1.19	1.06	1.16	1.25	1.40	1.28
