# Supplementary material for: A case-control study of trace-element status and lung cancer in Appalachian Kentucky
Source: PLoS One. 2019 Feb 27;14(2):e0212340. doi: 10.1371/journal.pone.0212340 (PMC6392268; doi:10.1371/journal.pone.0212340)
Supplement: S6 Table — (μg/L). (PDF) [file pone.0212340.s006.pdf]

S6 Table. Quintiles of drinking water trace element concentrations and method detection limits (μg/L).

Median Det Limit			Case/Control		Age				Smoking Status					
					<63		63+		Current		Former		Never	
			case	control	case	control	case	control	case	control	case	control	case	control
As	0.023	N	148	367	73	174	75	193	75	66	67	117	6	184
		50th	0.21	0.28	0.21	0.27	0.22	0.28	0.21	0.30	0.23	0.28	0.25	0.27
		90th	0.52	0.54	0.62	0.54	0.48	0.55	0.61	0.57	0.48	0.65	0.54	0.52
Cr	0.044	N	148	367	73	174	75	193	75	66	67	117	6	184
		50th	0.08	0.05	0.08	0.03	0.08	0.06	0.08	0.03	0.08	0.07	0.08	0.03
		90th	0.23	0.23	0.23	0.23	0.23	0.23	0.19	0.32	0.27	0.21	0.54	0.23
Ni	0.052	N	148	367	73	174	75	193	75	66	67	117	6	184
		50th	1.37	1.22	1.30	1.24	1.54	1.18	1.33	1.15	1.41	1.18	1.37	1.25
		90th	3.59	3.71	3.66	3.36	2.99	3.76	4.11	3.01	2.88	3.85	4.29	3.76
Cd	0.016	N	148	367	73	174	75	193	75	66	67	117	6	184
		50th	0.01	0.01	0.01	0.01	0.01	0.01	0.01	0.01	0.01	0.01	0.01	0.01
		90th	0.04	0.03	0.05	0.02	0.03	0.03	0.03	0.02	0.05	0.03	0.11	0.03
Pb	0.019	N	148	367	73	174	75	193	75	66	67	117	6	184
		50th	0.16	0.18	0.15	0.16	0.16	0.18	0.15	0.17	0.16	0.15	1.14	0.19
		90th	1.86	0.99	2.21	0.78	1.58	1.17	1.19	0.91	1.55	1.17	5.57	0.99
Zi	0.324	N	148	367	73	174	75	193	75	66	67	117	6	184
		50th	20.74	16.34	17.43	14.84	22.24	16.72	23.92	16.71	17.78	13.24	17.03	17.64
		90th	201.80	148.70	201.80	125.60	176.00	164.30	191.90	125.60	218.90	168.50	705.70	147.30
U	0.016	N	148	367	73	174	75	193	75	66	67	117	6	184
		50th	0.02	0.02	0.02	0.02	0.02	0.02	0.02	0.02	0.03	0.02	0.05	0.02
		90th	0.38	0.35	0.41	0.33	0.30	0.36	0.38	0.32	0.39	0.41	0.30	0.34
Fe	0.097	N	148	367	73	174	75	193	75	66	67	117	6	184
		50th	8.71	6.59	8.93	6.03	7.73	7.57	10.25	6.36	5.98	6.40	15.36	7.16
		90th	102.80	68.92	94.79	62.88	102.80	70.13	116.90	74.76	51.22	85.50	51.42	62.88
Al	0.545	N	148	367	73	174	75	193	75	66	67	117	6	184
		50th	27.00	21.86	36.07	15.93	20.42	26.66	17.93	23.47	44.16	32.69	22.46	15.31
		90th	153.90	142.30	170.00	119.70	122.40	153.80	112.40	163.80	186.90	169.20	110.10	119.70
Mn	0.027	N	148	367	73	174	75	193	75	66	67	117	6	184
		50th	0.59	0.50	0.59	0.46	0.59	0.53	0.64	0.54	0.59	0.48	0.36	0.53
		90th	11.28	5.59	5.24	4.61	11.28	5.81	27.67	19.23	3.28	5.40	3.79	4.52
Co	0.018	N	148	367	73	174	75	193	75	66	67	117	6	184
		50th	0.08	0.07	0.08	0.06	0.08	0.07	0.07	0.07	0.08	0.07	0.10	0.07
		90th	0.17	0.16	0.16	0.14	0.17	0.17	0.18	0.13	0.16	0.16	0.16	0.16
Cu	0.070	N	148	367	73	174	75	193	75	66	67	117	6	184
		50th	8.61	11.95	7.23	13.88	10.53	11.55	10.42	7.25	6.30	11.50	62.18	14.47
		90th	83.57	172.90	80.19	153.40	83.57	183.00	80.19	101.10	83.57	151.20	99.22	195.00

Median Det Limit			Case/Control		Age				Smoking Status					
					<63		63+		Current		Former		Never	
			case	control	case	control	case	control	case	control	case	control	case	control
**Se**	**0.082**	**N**	148	367	73	174	75	193	75	66	67	117	6	184
		**50th**	0.13	0.15	0.15	0.16	0.13	0.15	0.08	0.14	0.16	0.15	0.21	0.15
		**90th**	0.68	0.67	0.78	0.74	0.61	0.65	0.61	0.69	0.70	0.63	0.72	0.69
